# Alpha II Antiplasmin Deficiency Complicating Pregnancy: A Case Report

**DOI:** 10.1155/2011/698648

**Published:** 2011-05-15

**Authors:** Brenda Dawley

**Affiliations:** Department of Obstetrics and Gynecology, Joan C. Edwards School of Medicine, 1600 Medical Center Drive, Suite 4500 Huntington, WV 25701, USA

## Abstract

*Background*. Alpha II antiplasmin is a protein involved in the inhibition of fibrinolysis. A deficiency in this protein leads to increased hemorrhage. It is inherited in an autosomal recessive fashion. *Case*. 30-year-old Gravida 1, Para 0, presented for prenatal care with her first and subsequently her second pregnancy. Her medical history was significant for a known deficiency in alpha II antiplasmin. Her first and second pregnancies were complicated by nonobstetrical hemorrhage requiring transfusions and severe preeclampsia requiring preterm deliveries. *Conclusion*. Alpha II antiplasmin deficiency resulted in multiple episodes of nonobstetrical hemorrhages requiring transfusion and ultimately preterm deliveries due to severe preeclampsia. Both infants and mother had a good outcome. The presence of this disorder may require a multidisciplinary team approach involving obstetricians, pediatricians, and hematologists. *Precis*. Alpha II antiplasmin deficiency is a rare autosomal recessive disorder leading to increased fibrinolysis and hemorrhage. We present a case report of a pregnancy complicated by this disorder.

## 1. Introduction

Alpha II antiplasmin is a glycoprotein synthesized by the liver. It is present in two distinct forms: plasminogen binding and nonplasminogen binding. 20% of the plasminogen binding form is covalently linked to fibrin clots and renders them more resistant to lyses. It is inherited in an autosomal recessive fashion. A deficiency in its production allows the fibrin clots to lyse and dissolve prematurely resulting in hemorrhage. It is also a strong inhibitor of plasmin, an enzyme directly involved in fibrinolysis [1]. 

Plasmin is involved in the cleavage of extracellular vascular endothelial growth factor. A deficiency in its inhibitor (alpha II antiplasmin) has been shown in vitro mice studies to increase the mortality from an acute myocardial infarction. VEGF has been shown to increase the vascular permeability in ischemic tissue; its overproduction results in pulmonary edema and increased mortality. Elevated levels of VEGF have been associated with increased incidence of preeclampsia [2].

## 2. Case

Our patient first presented to our service at 10-week gestation. She was a 30-year-old Caucasian female Gravida 1, Para zero. She had been diagnosed with alpha II antiplasmin deficiency with a level 50% normal due to several episodes of severe hematuria secondary to nephrolithiasis. She had received multiple blood transfusions in her adolescence due to the severity of these bleeds. Her brother had been diagnosed with alpha II antiplasmin deficiency and Von Willebrand's disease; the patient had tested negative for Von Willebrand's disease. Consanguinity was denied by the patient.

Her past surgical history was significant for a laparoscopic excision of a benign ovarian cyst and a tonsillectomy. She was single, nonsmoker, and nondrinker and denied illicit drug use. Her family history was significant for the brother with the bleeding diathesis. She also had a cousin diagnosed with Trisomy 21. Her father and her maternal and paternal grandmothers suffered from hypertension.

With her first pregnancy, she presented at 10-week gestation with recurrent nephrolithiasis and hematuria. Her hemoglobin was 9.6; she was controlled with intravenous fluids and narcotics for pain control; she was discharged after 5 days and represented one week later with similar complaints.

Nephrology was consulted; 24-hour urine revealed excess urine calcium excretion of 582 mg/day. She was begun on amiloride 5 mg daily to control her hypercalcuria. She was readmitted at 17-week gestation with frank hematuria and passage of large clots of blood in her urine and severe back pain. Her hemoglobin remained stable at 9.6; conservative management continued. A PICC line was required to maintain intravenous access. She was discharged after several days and remained as an outpatient until 25-week gestation when she presented with heavy vaginal bleeding that soaked at least 4 peripads. She received 2-unit fresh frozen plasma and her bleeding resolved. A negative fetal cell stain and normal ultrasound revealed an appropriately sized infant with a normally implanted placenta. She received a course of betamethasone due to preterm status. She was observed for several days and was discharged home with the diagnosis of a stable placenta abruption. Close outpatient follow-up with serial nonstress tests and biophysical profiles was initiated. She remained stable until 32-week gestation when recurrent vaginal bleeding developed. She was hospitalized and at 34-week gestation oligohydramnios and new onset hypertension developed. She was ultimately diagnosed with severe preeclampsia by blood pressure criteria; she was begun on magnesium sulfate for seizure prophylaxis and cervidil and pitocin induction. She had a spontaneous vaginal delivery complicated by postpartum hemorrhage that responded to fresh frozen plasma. Her infant was a female weighing 1760 grams with agars 6 at 1 minute and 8 at 5 minutes. Her prenatal course was complicated by hyermagnesemia, hyperbilirubinemia, and respiratory depression; she required transient oxygen support and was discharged on day of life 8.

Our patient represented with her second pregnancy at 8-week gestation. She had required hospitalization several times in the interim due to hematuria and nose bleeds. Her second pregnancy was complicated by fetal bilateral choroid plexus cysts and recurrent migraines. She declined genetic amniocentesis and remained an outpatient until 30-week gestation. She presented with massive nasal bleeding requiring transfusion of fresh frozen plasma and 2-unit packed red blood cells. She was also begun on aminocaprioc acid 5 grams every 6 hours until her bleeding resolved. She required surgical intervention by EENT. She developed elevated blood pressure and oligohydramnios requiring induction of labor and magnesium sulfate prophylaxis and delivered at 33-week gestation. She had received betamethasone prior to delivery. She delivered a viable male weighing 1980 grams with agars 9 at one minute and 9 at 5 minutes. His neonatal course was complicated by apnea, feeding difficulties, respiratory distress syndrome, and hyperbilirubinemia. He was discharged on day of life 21.

## 3. Discussion

Alpha II antiplasmin deficiency is a rare autosomal recessive disorder leading to increased lyses of fibrin clots and increased risk hemorrhage. When blood clots, multiple factors including plasminogen are bound to fibrin: this complex leads to plasminogen activation and conversion to plasmin. Plasmin digests the fibrin component of the thrombus. Inhibition of this process is achieved mainly through several inhibitors including plasminogen activator inhibitor and alpha II antiplasmin inhibitor. The plasmin and alpha II antiplasmin form a stable, inactive complex preventing further lyses of fibrin. Lack of alpha II antiplasmin will lead to increased levels of plasmin and increased fibrinolysis and hemorrhage. 

Our patient experienced multiple episodes of bleeding; control required use of both fresh frozen plasma and aminocaprioc acid. Fresh frozen plasma is effective in control of bleeding by replacing the fibrinogen and allowing normal clot formation to continue. Aminocaprioc acid inhibits fibrinolysis of the fibrin plug. Contra indications include sensitivity to drug, disseminated intravascular coagulopathy, and severe renal, hepatic, or cardiac disease. 33% of patients will experience significant gastrointestinal side effects such as nausea, abdominal pain, and diarrhea. It is considered an FDA class C drug in pregnancy.

 Levels of the alpha II antiplasmin-plasmin complex has been shown to be elevated in acute stroke and myocardial infarction. Lack of alpha 2 antiplasmin in mice has been shown to increase the mortality after an acute myocardial infarction [3]. Alpha 2 antiplasmin is essential in the regulation of circulating vascular endothelial growth factor; a deficiency is associated with increased levels of VEGF. Increased levels of VEGF post myocardial infarction appears associated with increased levels of cell damage and mortality [2]. VEGF has recently been shown to be involved in the placental implantation process; elevated levels in early pregnancy have been associated with increased risk of developing preeclampsia. Our patient developed preterm severe preeclampsia with both her pregnancies. The elevated level of VEGF resulting from her alpha II antiplasmin deficiency may have been involved in this process. Unfortunately, her level of VEGF was not determined at the time of her pregnancy. Further areas of study may involve determining the level of alpha II antiplasmin in the first trimester of pregnancy to determine if higher levels are associated with an increased risk of developing preeclampsia [4]. If higher levels are discovered, implementation of close follow-up and observation of increased edema and increased vascular resistance may allow the prevention of preeclampsia.
